# Comparative Analysis of Machine Learning Models for Nanofluids Viscosity Assessment

**DOI:** 10.3390/nano10091767

**Published:** 2020-09-07

**Authors:** Mohammadhadi Shateri, Zeinab Sobhanigavgani, Azin Alinasab, Amir Varamesh, Abdolhossein Hemmati-Sarapardeh, Amir Mosavi, Shahab S

**Affiliations:** 1 Department of Electrical & Computer Engineering, McGill University, Montreal, QC H3A 2K6, Canada; mohammadhadi.shateri@mail.mcgill.ca (M.S.); zeinab.sobhanigavgani@mail.mcgill.ca (Z.S.); azin.alinasab@polymtl.ca (A.A.); 2 Department of Chemical & Petroleum Engineering, University of Calgary, Calgary, AB T2N 1N4, Canada; amir.varamesh@ucalgary.ca; 3 Department of Petroleum Engineering, Shahid Bahonar University of Kerman, Kerman 7616913439, Iran; 4 College of Construction Engineering, Jilin University, Changchun 130600, China; 5 Faculty of Civil Engineering, Technische Universität Dresden, 01069 Dresden, Germany; 6 School of Economics and Business, Norwegian University of Life Sciences, 1430 Ås, Norway; 7 Institute of Automation, Kando Kalman Faculty of Electrical Engineering, Obuda University, 1034 Budapest, Hungary; 8 Institute of Research and Development, Duy Tan University, Da Nang 550000, Vietnam; 9 Future Technology Research Center, College of Future, National Yunlin University of Science and Technology, 123 University Road, Section 3, Douliou, Yunlin 64002, Taiwan

**Keywords:** nanofluid viscosity, experimental data, machine learning, deep learning, nano, nanomaterials, nanofluid, artificial neural network, data science, big data, ensemble models, artificial intelligence, computational fluid dynamics, material design, computational mechanics

## Abstract

The process of selecting a nanofluid for a particular application requires determining the thermophysical properties of nanofluid, such as viscosity. However, the experimental measurement of nanofluid viscosity is expensive. Several closed-form formulas for calculating the viscosity have been proposed by scientists based on theoretical and empirical methods, but these methods produce inaccurate results. Recently, a machine learning model based on the combination of seven baselines, which is called the committee machine intelligent system (CMIS), was proposed to predict the viscosity of nanofluids. CMIS was applied on 3144 experimental data of relative viscosity of 42 different nanofluid systems based on five features (temperature, the viscosity of the base fluid, nanoparticle volume fraction, size, and density) and returned an average absolute relative error (AARE) of 4.036% on the test. In this work, eight models (on the same dataset as the one used in CMIS), including two multilayer perceptron (MLP), each with Nesterov accelerated adaptive moment (Nadam) optimizer; two MLP, each with three hidden layers and Adamax optimizer; a support vector regression (SVR) with radial basis function (RBF) kernel; a decision tree (DT); tree-based ensemble models, including random forest (RF) and extra tree (ET), were proposed. The performance of these models at different ranges of input variables was assessed and compared with the ones presented in the literature. Based on our result, all the eight suggested models outperformed the baselines used in the literature, and five of our presented models outperformed the CMIS, where two of them returned an AARE less than 3% on the test data. Besides, the physical validity of models was studied by examining the physically expected trends of nanofluid viscosity due to changing volume fraction.

## 1. Introduction

Conventional working mediums, such as water, ethylene glycol, etc., cannot provide enough efficiency in industrial processes since they suffer from relatively poor heat transfer characteristics. Investigations have proved that the suspension of solid particles into traditional fluids enhances their heat transfer capability. Based on this idea and with advances in nanotechnology, a new generation of heat transfer fluid, named as nanofluids, was invented. Nanofluids are the suspension of nanoparticles (such as metals, metal oxides, non-metals, etc.) whose dimensional size is typically in the range of 1–100 nm in a base fluid (such as water, oil, ethylene glycol, etc.) [1,2]. In comparison to conventional working mediums, nanofluids can provide much more efficient thermo-physical properties, which are the controlling factors for flow behavior and heat transfer ability of working fluids [3,4,5,6]. Therefore, the thermo-physical properties of nanofluids, including viscosity, are important parameters that must be evaluated before any application of them.

The viscosity of fluids is a measure of its resistance to flow. Nanofluids viscosity directly impacts the required pumping power and associated pressure drop in any energy system. In addition, the amount of heat augmentation in convection is strictly influenced by nanofluid viscosity. Moreover, the viscosity of nanofluids should be determined accurately since the value of critical dimensionless numbers, including Brinkman number, Prandtl number, and Reynolds number, are related to the value of viscosity [7,8]. Considering the importance of the viscosity of nanofluids, over the past years, different methods and equations were introduced to predict that. Einstein [9] was the first one who proposed an equation for the estimation of the viscosity of floating small particles with low volume fraction into a fluid. After Einstein’s [9] pioneering work, many other authors tried to propose new methods for the prediction of viscosity of suspended particles, either by modifying Einstein’s [9] equation or developing a new method. Brinkman [10], Lundgren [11], Frankel and Acrivos [12], Batchelor [13], Thomas and Muthukumar [14], Chen et al. [15], Maiga et al. [16] are among the most well-known developed models. A recent review of the available models for the prediction of viscosity of nanofluids was published by Varamesh and Hemmati-Sarapardeh [17]. They reviewed almost all the important models for the viscosity of nanofluids and categorized them into three main types, theoretical models, empirical equations, and computer-aided models.

Recently, artificial intelligent models, such as an artificial neural network (ANN), radial basis function neural network (RBF-NN), etc., which have powerful nonlinear regression ability and can theoretically model complex relations, have been widely utilized to model thermo-physical properties of nanofluids. These strong data-driven modeling tools can determine the complex nonlinear dependency of an output parameter to its input variables with high speed and low computational cost [6,18]. Karimi et al. [19] firstly proposed an artificial neural network based on a genetic algorithm (GA). They gathered 381 experimental data from eight different types of nanofluids for the development of the model by considering the input variables, including particle volume concentration, temperature, the viscosity of fluid base, particle size, and density ratio of base fluid to the nanoparticle. Their statistical results showed that the predicted values and experimental data were in good agreement. The mean average relative error of the model was 2.48%. Since then, several other computer-aided data-driven models have been developed by considering different intelligent modeling approaches, including fuzzy C-means clustering-based adaptive neuro-fuzzy system (FCM-ANFIS), hybrid self-organizing polynomial neural networks (PNN) based on group method of data handling (GMDH), least-square support vector machine (LSSVM), radial basis function neural networks (RBF-NN), genetic algorithm-polynomial neural network (GA-PNN), multilayer perceptron neural networks (MLP-NNs), gene expression programming (GEP) [8,20,21,22,23,24,25,26,27,28,29,30,31,32,33,34,35]. However, the most accurate model with a wide range of applicability for the prediction of viscosity of nanofluids was developed by Hemmati-Sarapardeh et al. [1], based on a committee machine intelligent system (CMIS). They introduced a machine learning model based on the combination of seven baselines, including four MLP-NNs, two RBF-NNs, and an LSSVM. Each of the MLP-NNs was optimized with different algorithms, including Levenberg–Marquardt (LM), resilient backpropagation (RB), Bayesian regularization (BR), and scaled conjugate gradient (SCG). The RBF-NNs were optimized by utilizing particle swarm optimization (PSO) and the genetic algorithm (GA). The LSSVM model was optimized with coupled simulated annealing (CSA). The proposed CMIS by Hemmati-Sarapardeh et al. [1] was applied on 3144 experimental data of relative viscosity of 42 different nanofluid systems by considering temperature, the viscosity of the base fluid, nanoparticle volume fraction, size, and density as input variables to predict relative viscosity as the output variable. The obtained results by Hemmati-Sarapardeh et al. [1] showed good agreement with experimental data with an average absolute relative error of 3.95% between predicted relative viscosity values and corresponding experimental data. Besides, the developed CMIS outperformed all of the investigated available models, and unlike the previously available models, the developed CMIS showed high accuracy over the whole range of input variables. Hemmati-Sarapardeh et al. [1] also analyzed the quality of the gathered 3144 experimental data points and showed that all of the data points had very good reliability except a small percent of them.

This study aimed to improve the accuracy and efficiency of the CMIS model developed by Hemmati-Sarapardeh et al. [1], which is the best available model. For this purpose, in this study, using the same data set gathered by Hemmati-Sarapardeh et al. [1], the two main baselines used by them, including a multilayer perceptron (MLP) network and LSSVM, were considered, and the hyperparameters of each were tuned. Moreover, a deeper MLP network, decision tree (DT) model, a random forest (RF) model, and extra trees model were used to improve the performance of the CMIS model developed by Hemmati-Sarapardeh et al. [1].

## 2. Data Collection

In this study, the most comprehensive data bank of nanofluid viscosity was used to develop reliable and accurate models. This data bank, which was already used in our previous study [1], covers 3144 experimental data points of nanofluid viscosity of 42 various types of nanofluid samples. Nanoparticle size, temperature, particle volume fraction, the density of the nanoparticles, and the viscosity of the base fluid were selected as the inputs of models, whereas the relative viscosity of nanofluid was assumed as the output. Details for the used data are summarized in Table 1. For more information about this data bank, readers can refer to our previous study [1]. The reason why the number of data points for some particles was low is that there are only limited studies regarding the evaluation of the viscosity of these particles, and we could not find more experimental data for these particles.

## 3. Model Development

In this section, all the models used in this study are introduced.

### 3.1. Multilayer Perceptron Network

**Figure 1:**
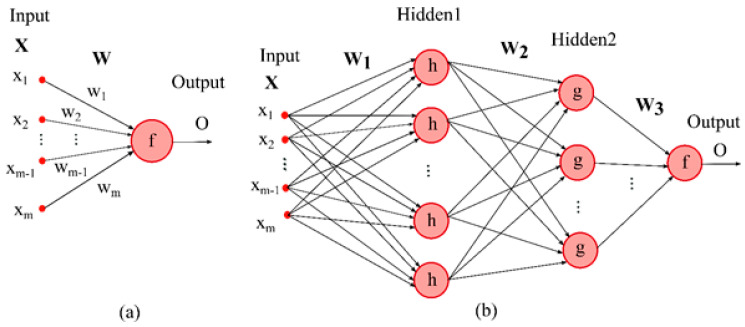
(**a**) Perceptron model, (**b**) Multilayer perceptron (MLP) with two hidden layers (Note that f, g, and h are activation functions, while the w represents the weights that the model needs to learn. x is also input of the model).

The perceptron learning rule introduced by Rosenblatt [74] corresponds to a simple model consisting of one neuron (illustrated in Figure 1a) in which the output is a function of the sum of weighted inputs modified by an activation function or transfer function (f).

The multilayer perceptron (MLP) is a system of interconnected perceptron models. It is proved that the MLP can be trained to model every smooth, measurable function without concerning the data distribution [75]. In other words, using nonlinear activation functions gives the MLP networks the ability to approximate non-linear functions, while MLP with linear activation function can only model linear functions. For MLP networks, four kinds of hyperparameters, including the type of activation functions used in hidden and output layers, number of hidden layers, number of neurons in each hidden layer, and optimizer method, can be assumed and need to be determined before using the network for prediction. Tanh and Sigmoid are the most commonly used activation functions used in MLPs [76]. The optimizer has a pivotal role in the performance of MLP. In this study, four types of optimizers were used, and each of them has been explained in the next section.

### 3.2. Support Vector Machine for Regression

The support vector machine (SVM) was largely developed at AT&T Bell Laboratories. The support vector machine for regression (SVR) was first introduced in 1997 by H. Drucker et al. [77], following Cortes and Vapnik’s work on support vector machines (SVM) [78]. SVR uses the same principles as SVM, with only a few minor differences. The SVR is a method to estimate a function that maps the input to a continuous number, which is the target output. SVM for classification does not apply a penalty on the points far away from the hyperplane as long as the class is predicted correctly; however, SVR needs the estimated function to be as close as possible to all target points. Therefore, SVR will apply a penalty on all points out of a predefined margin, *ε*, from the estimated function. In other words, SVR does not care about the errors as long as they are less than *ε*, but will not accept any deviations higher than *ε*. In the case of a linear function, f(X)=w·x+b, we can describe the problem as a convex optimization problem:

(1) $$minimize\;\frac{1}{2}{∥w∥}^{2}\;subject\;to\;\{\begin{array}{c} y_{i}-w\cdot x_{i}-b\leq \varepsilon \\ w\cdot x_{i}+b-y_{i}\leq \varepsilon \end{array}$$

where $y_{i}$ represents the true value of the *i*th sample, while the w and b are the weight and bias associated with the model, respectively. The value *x* is also input of the model.

**Figure 2:**
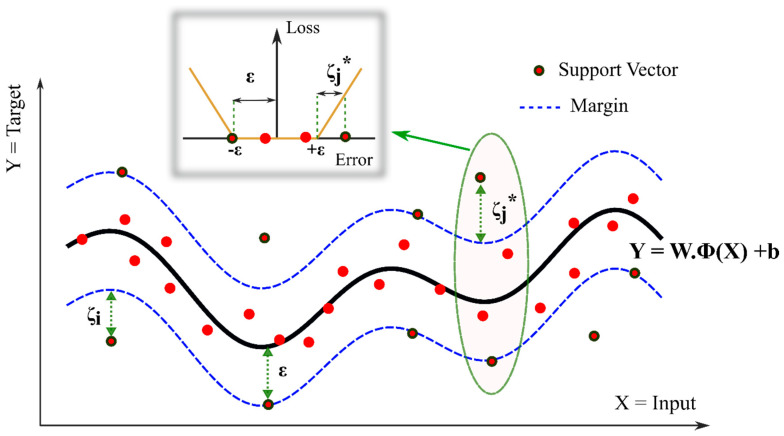
Soft margin nonlinear SVR (support vector machine for regression) with ε-insensitive loss function (*ϕ*(.) is a feature mapping function, *W* is the weight matrix, and *b* is bias vector).

Soft SVM: Similar to SVM, one can modify the optimization loss function by introducing slack variables, *ζ_i_* and ζ_i_^*^, to allow a soft margin when finding a function that can map all samples within the predefined margin, which is not feasible. The optimization problem will change as follows:

(2) $$minimize\;\frac{1}{2}{∥w∥}^{2}+C\overset{l}{\underset{i=1}{\sum }}\left(\zeta_{i}+\zeta_{i}^{*}\right)\;subject\;to\;\{\begin{array}{l} y_{i}-w\cdot x_{i}-b\leq \varepsilon +\zeta_{i}\\ w\cdot x_{i}+b-y_{i}\leq \varepsilon +\zeta_{i}^{*}\\ \zeta_{i},\zeta_{i}^{*}\geq 0 \end{array}$$

where *C* ≥ 0 is the variable that specifies how much deviation more than *ε* is allowed. Greater *C* is closer to hard margin SVR. This corresponds to introducing the *ε*-insensitive loss function. Figure 2 shows the concept of soft margin and the ε-insensitive loss function.

(3) $${\left|\zeta \right|}_{\varepsilon }=\{\begin{array}{ll} 0 & if\;\left|\zeta \right|\leq \varepsilon \\ \left|\zeta \right|-\varepsilon & otherwise \end{array}$$

The quadratic optimization problem (2) can be modeled and solved in two different ways, including primal and dual.

Primal: Introducing Lagrangian multipliers $\eta_{i}$, $\eta_{i}^{*}$, $\alpha_{i}$, $\alpha_{i}^{*}$, the Equations (4) and (5) show the primal form of the optimization problem:

(4) $$\begin{array}{l} minimize\;\frac{1}{2}{∥w∥}^{2}+C\overset{l}{\underset{i=1}{\sum }}\left(\zeta_{i}+\zeta_{i}^{*}\right)-\overset{l}{\underset{i=1}{\sum }}\left(\eta_{i}\zeta_{i}+\eta_{i}^{*}\zeta_{i}^{*}\right)\\ \;\;\;\;\;\;\;-\overset{l}{\underset{i=1}{\sum }}\alpha_{i}\left(\varepsilon +\eta_{i}+y_{i}+w\cdot x_{i}+b\right)\\ \;\;\;\;\;\;\;-\overset{l}{\underset{i=1}{\sum }}\alpha_{i}^{*}\left(\varepsilon +\eta_{i}^{*}+y_{i}+w\cdot x_{i}+b\right)\;subject\;to\;\{\begin{array}{c} \alpha_{i},\alpha_{i}^{*}\geq 0\\ \eta_{i},\eta_{i}^{*}\geq 0 \end{array}\; \end{array}$$

Dual: The dual form of the optimization problem can be derived using the primal objective as below:

(5) $$\begin{array}{c} maximize\;\overset{l}{\underset{i=1}{\sum }}y_{i}\left(\alpha_{i}-\alpha_{i}^{*}\right)-\varepsilon \overset{l}{\underset{i=1}{\sum }}\left(\alpha_{i}+\alpha_{i}^{*}\right)-\frac{1}{2}\overset{l}{\underset{i=1}{\sum }}\overset{l}{\underset{j=1}{\sum }}\left(\alpha_{i}-\alpha_{i}^{*}\right)\left(\alpha_{j}-\alpha_{j}^{*}\right)x_{i}x_{j}\\ subject\;to\;\{\begin{array}{l} \overset{l}{\underset{i=1}{\sum }}\left(\alpha_{i}-\alpha_{i}^{*}\right)=0\\ 0\leq \alpha_{i},\alpha_{i}^{*}\leq C \end{array} \end{array}$$

Kernels: When the data is not linearly separable (classification), one can use the kernel functions to transform the data into a higher dimensional feature space, where it turns to a linearly separable data. For the regression case, non-linear SVR can be done by kernelizing the data. Common kernel functions are summarized as follows:

(6) $$\{\begin{array}{l} Polynomial:\;k\left(x,x^{\prime }\right)={\left(<x,x^{\prime }>+c\right)}^{d}\\ Tanh:\;k\left(x,x^{\prime }\right)=tanh\left(\theta +\emptyset <x,x^{\prime }>\right)\\ RBF:k\left(x,x^{\prime }\right)=exp\left(\frac{{∥x-x^{\prime }∥}^{2}}{2\sigma^{2}}\right) \end{array}$$

### 3.3. Decision Tree

**Figure 3:**
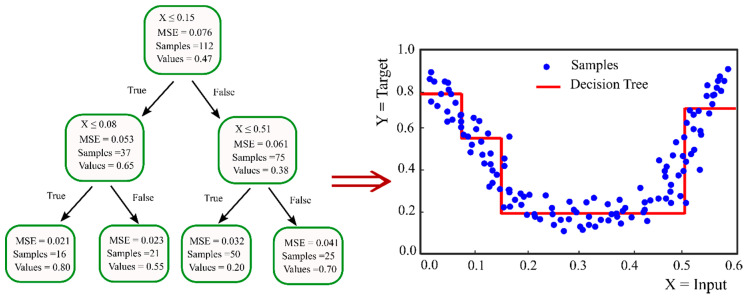
Examples of using a decision tree for regression.

A decision tree that can be used for both regressions and classification problems is a non-parametric supervised learning algorithm. Messenger and Mandell in 1972 [79] proposed the first classification tree algorithm, which was named as THAID. The decision tree is a hierarchical tree-like flowchart composed of a root node, internal nodes, leaf nodes, and branches. The topmost node with no incoming branch, which represents the entire sample space, is called the root node. The nodes with one incoming branch and two or some outgoing edges are known as internal or test nodes, and all other nodes representing the final results are leaves, also named as terminal nodes. Splitting, stopping, and pruning are the main steps for building a decision tree [80]. Splitting the data means recursively partitioning the input data into two or more subsets based on testing the most significant attribute, which can separate the training instances as well as possible. The significant attribute is determined by different criteria, including the Gini index, entropy, classification error, gain ratio, information gain, and towing [81] for classification trees, and variance reduction or standard deviation-reduction for regression trees. Figure 3 represents an example of a decision tree for classification and regression problems. Splitting the data starts from the root node and continues on internal nodes until predefined homogeneity or stopping criteria is satisfied. Determining the stopping criteria, such as the minimum number of records in a node prior to splitting, the minimum number of records in a leaf, and the depth of any leaf from the root node, reduces the complexity of the tree, which prevents overfitting. Without stopping criteria, the splitting continues until a complex tree is constructed, in which the records in each node are 100% pure; such a tree would be fitted very well on the training data, but will not perform well on the unseen data. Therefore, these stopping criteria are normally tuned during training the model to choose the best values. Pruning is another method to avoid overfitting when stopping methods do not perform satisfactorily. In pruning, a complete tree is grown and then pruned back to a smaller tree by eliminating nodes, which have less information gain on the validation set.

### 3.4. Random Forest and Extra Trees

Random forest (RF) is an ensemble method, covering both classification and regression tasks. This algorithm creates a forest of multiple decision trees. The random forest algorithm, proposed by Leo Breiman [82], combines Breiman’s idea of bagging with the random decision forests algorithm developed by Tin Kam Ho [83]. The random forest uses the randomly created trees approach. For creating each tree of the forest, k features of total m features are selected randomly (where k < m), the best feature is chosen by the splitting methods for the root node, and the internal nodes tests are selected by the same splitting approach until reaching the leaves. To predict unseen data with the trained model, each tree predicts and stores the outcome. The final forest output will be the majority voted class by different classification trees in case of classification, or the average of outcomes by different regression trees in case of regression. Random forest robustness against overfitting is one of the most important advantages of this algorithm, comparing the decision tree [84]. There is another tree-based ensemble model, which is called the extra trees (ET) algorithm. In contrast to the RF, which tests all possible splits over a fraction of features, the ET method tests random splits (cut point) over a fraction of features [85]. Besides accuracy, the ET method is computationally efficient compared to the RF. In this study, both methods were used and compared with other methods.

### 3.5. Optimization Methods

The gradient descent (GD) algorithm is one of the most well-known optimization methods. Considering the objective function *J*(*θ*) parameterized by model parameter *θ*, the GD updates the *θ* in the opposite direction of the gradient of *J*(*θ*) as follows:

(7) $$\theta =\theta -\eta \cdot \nabla_{\theta }J\left(\theta \right)$$

where *η* is the learning rate. There are other versions of GD called stochastic gradient descent (SGD) and mini-batch gradient descent, which are faster than SGD [86]. Choosing proper learning rate, learning rate schedule, and trapping in (escaping) the local minima are some challenges with SGD and its other versions. To address these challenges, the following optimization algorithms that are extensively used in deep learning are provided.

Momentum: When the SGD method encounters ravines, which are common around local optima, it starts to oscillate across the slope of the ravine. The momentum method [87] helps the SGD to damp the acceleration toward the local optima in the true direction by adding a fraction of previous updates.

(8) $$\{\begin{array}{l} v_{t}=\gamma v_{t-1}+\eta \cdot \nabla_{\theta }J\left(\theta \right)\\ \theta =\theta -v_{t} \end{array}$$

where γ is called the momentum term.

NAG: Nesterov accelerated gradient or NAG is similar to the momentum method, except it calculates the gradient with regards to the future position of the parameter as follows [88]:

(9) $$\{\begin{array}{l} v_{t}=\gamma v_{t-1}+\eta \cdot \nabla_{\theta }J\left(\theta -\gamma v_{t-1}\right)\\ \theta =\theta -v_{t} \end{array}$$

Adagrad: Adaptive gradient algorithm (Adagrad), introduced by Duchi et al. [89], modifies the learning rate η for parameter θ*_i_* at time step *t* using the past gradients used for θ*_i_* as follows:

(10) $$\theta_{t+1,i}=\theta_{t,i}-\frac{\eta }{\sqrt{G_{t,ii}+\varepsilon }}g_{t,i}$$

where $g_{t,i}=\nabla_{\theta_{i}}J\left(\theta_{t,i}\right)$ and *G_t_* is a diagonal matrix, where each diagonal element ii is the sum of squared gradients with regards to *θ_i_* up to time step t.

Using this modification, the infrequent parameters have a larger update, while there is a smaller update for the frequent parameter. The Adagrad has been used in different learning applications; however, the accumulation of the squared gradients in the denominator of the learning rate eventually shrinks the learning rate and makes the update rule ineffective.

Adadelta: To address the issue mentioned in Adagrad, the Adadelta method is introduced by Zeiler [90]. The main idea is that, instead of accumulating all the past squared gradients, just a fixed number of them, *w*, are used. This prevents the monotonical shrinkage of the learning rate. In practice, to avoid storing w previous squared gradients, an exponentially decaying average of squared gradients is used as follows:

(11) $$\{\begin{array}{l} E{\left(g^{2}\right)}_{t}=\eta E{\left(g^{2}\right)}_{t-1}+\left(1-\eta \right){g_{t}}^{2}\;\\ \Delta \theta_{t}=\frac{RMS{\left[\Delta \theta \right]}_{t-1}}{RMS{\left[g\right]}_{t}}\cdot g_{t} \end{array}$$

where $RMS{\left[x\right]}_{t}=\sqrt{E{\left(x^{2}\right)}_{t}+\varepsilon }$ for any x.

Adam: Adaptive moment (Adam) is another optimization method, which is a combination of the momentum method and Adadelta [91]. This method uses the exponentially decaying average of previously squared gradients (similar to Adadelta) and the exponentially decaying average of gradients (similar to momentum).

(12) $$\theta_{t+1}=\theta_{t}-\frac{\eta }{\sqrt{{\hat{v}}_{t}}+\varepsilon }{\hat{m}}_{t}$$

where ${\hat{m}}_{t}=\frac{m_{t}}{1-{\beta_{1}}^{t}},\;{\hat{v}}_{t}=\frac{v_{t}}{1-{\beta_{2}}^{t}},\;m_{t}=\;\beta_{1}m_{t-1}+\left(1-\beta_{1}\right)g_{t}$ and $v_{t}=\beta_{2}v_{t-1}+\left(1-\beta_{2}\right){g_{t}}^{2}$.

AdaMax: AdaMax is a variant of the Adam method, which scales the gradients based on the infinite norm, instead of the *l*_2_ norm [91].

(13) $$\theta_{t+1}=\theta_{t}-\frac{\eta }{u_{t}}{\hat{m}}_{t}$$

where $u_{t}={\beta_{2}}^{\infty }v_{t-1}+\left(1-{\beta_{2}}^{\infty }\right){g_{t}}^{\infty }=\max\left(\beta_{2}v_{t-1},\;\left|g_{t}\right|\right)$.

Nadam: Nesterov accelerated adaptive moment (Nadam) is a combination of the Adam and NAG methods [92].

(14) $$\theta_{t+1}=\theta_{t}-\frac{\eta }{\sqrt{{\hat{v}}_{t}}+\varepsilon }\left(\beta_{1}{\hat{m}}_{t}+\frac{\left(1-\beta_{1}\right)g_{t}}{1-{\beta_{1}}^{t}}\right)$$

In this work, four of these optimizers, including Adagrad, Adadelta, AdaMax, and Nadam, were used in optimizing the MLP network parameters.

## 4. Results and Discussion

Generally, two kinds of baseline models, including an MLP network with two hidden layers and an LSSVM model, were used in [1]. In this part, hyperparameters of these two baselines were tuned using validation data set for better performances. More specifically, the data sets were split into train and test sets with a ratio of roughly 85:15, while 10% of the training data was used as the validation set, which was used to set the hyperparameters. In this work, the MLP network with two hidden layers and four different optimizers was used as a baseline in [1]. In all of them, the Tanh, Sigmoid were used as activation functions for first and second hidden layers, respectively. Moreover, in all of the MLP networks, pure linear activation was used for the output layer. Among all of these MLP networks, MLP with Bayesian regularization (BR) optimizer returned the lowest average absolute relative error (AARE) of 4.931% on the test data. We believed by tuning the number of neurons in each hidden layer and selecting an appropriate optimizer method, the performance of MLP could be improved. Another option for improving the performance of MLP was increasing the number of hidden layers (deeper MLP), and we have used this option in the next section as one of our suggested models. Therefore, in this work, the same architecture, as [1], was used, but the number of neurons in each hidden layer and the type of optimizer were tuned as a validation set using a grid search method. As the preprocessing, the input data were normalized, i.e., each feature was centered by its mean value and scaled by its standard deviation. It should be noted that in this work, the MLP form was generally considered as (Input: number of neurons)(Hidden 1: number of neurons, activation function)(Hidden l: number of neurons, activation function)(Output: number of neurons, activation function)-optimizer. The result of the Grid search showed that MLP with form (5)(Tanh,32)(Sigmoid,64)(Linear,1)-Nadam had the best performance on the validation data set. Another modification that might improve the performance of the MLP used in [1] was the type of activation function. More specifically, in their MLP, the output layer had a pure linear activation function, and if be replaced with a non-linear activation function, they might improve the performance. Therefore, MLP with sigmoid activation function at the output layer was tuned for the appropriate number of neurons in hidden layers and type of optimizer. The Grid search result returned MLP with the form (5)(Tanh,32)(Sigmoid,64)(Sigmoid,1)-Nadam as the best model. Another baseline used in [1] was the least square SVM (LSSVM), which returned an AARE of 6.630% on the test data. In this work, the SVM regression with radial basis function (RBF) kernel was used. Our assumption was that since Gaussian models are the most commonly used models for real-world data, Gaussian kernel should be used in SVM. Two hyperparameters, including penalty parameter C and kernel coefficient gamma, were considered and tuned using the Grid search algorithm. The result showed that RBF-SVM with C = 1 and gamma = 2.3 provided the lowest AARE on validation data. Other models, including decision tree, random forest, extra trees, and an MLP with three hidden layers, could be used to improve the performance of the CMIS model used as the main model in [1]. The results of the CMIS model on different ranges of input features showed that in some ranges (for example, range 50–75 for nanoparticle size), the model had poor performance (large AARE), while in some other ranges, it had better performance. This behavior suggested that algorithms, such as decision tree (DT) or an ensemble of them (random forest and extra trees), which goes through each feature and finds the best test split based on the different ranges of features, might be a good candidate. To this end, four hyperparameters, including a maximum number of features, maximum depth, the minimum number of sample split, and a minimum number of leaf nodes, were considered and tuned using Grid search. The results of the Grid search suggested a DT with maximum depth equal to14, the maximum number of features equal to 4, the maximum number of leaf nodes of 450, and the minimum number of sample split of 3. As another attempt to improve the results, random forest (RF) was used as an ensemble of DTs. Hyperparameters, including a maximum number of features and the maximum depth, were tuned using the Grid search, and the results based on the best parameters maximum depth 18 and a maximum number of features 4 were provided. Extra trees (ET) was another ensemble of DTs. Similar to the DT, four hyperparameters were tuned, and the optimum values were the maximum depth of 20, the maximum number of features of 5, the maximum number of leaf nodes of 1000, and the minimum number of sample split of 4. The MLP network with three hidden layers was the last model used to improve the performance. Similar to the MLP with two hidden layers, hyperparameters (including the number of neurons in hidden layers and the type of optimizer) of MLP with three hidden layers were tuned using the Grid search. Again, the last activation function was changed to a Sigmoid function to see whether any improvement had been achieved. Table 2 lists the performance of each mentioned model using statistical parameters, including average absolute relative error (AARE (%)), average relative error (ARE (%)), root mean square error (RMSE), and standard deviation (SD).

**Figure 4:**
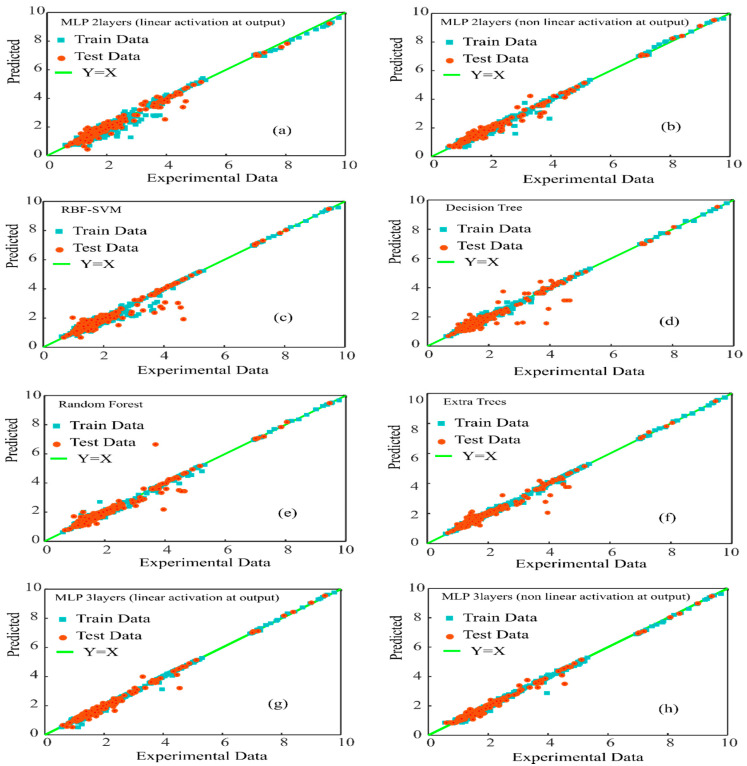
Cross plot of predicted viscosity versus experimental value, (**a**): MLP 2 layers (linear activation at output), (**b**): MLP 2 layers (non linear activation at output), (**c**): RBF-SVM, (**d**): Decision Tree, (**e**): Random Forest, (**f**): Extra Trees, (**g**): MLP 3 layers (linear activation at output), (**h**): MLP 2 layers (non linear activation at output).

From this table, it can be seen that the baselines model SVM and two layers MLP compared to baselines [1] were improved by fine-tuning. In addition, the suggested models, including random forest (RF), extra trees (ET), and three layers MLP (both with and without nonlinear activation at the output layer), provided better performance than the CMIS model. To see the performance of the models visually, the cross plot of the training and test data for each model are represented in Figure 4. In this figure, closer data points to the unit slope line indicated a better prediction of viscosity. For example, comparing Figure 4a,b, it can be seen how a nonlinear activation at the output layer improved the performance of MLP, or by comparing the trees-based methods in Figure 4d–f, the RF and ET outperformed the DT by providing more prediction close to the unit slope line.

**Figure 5:**
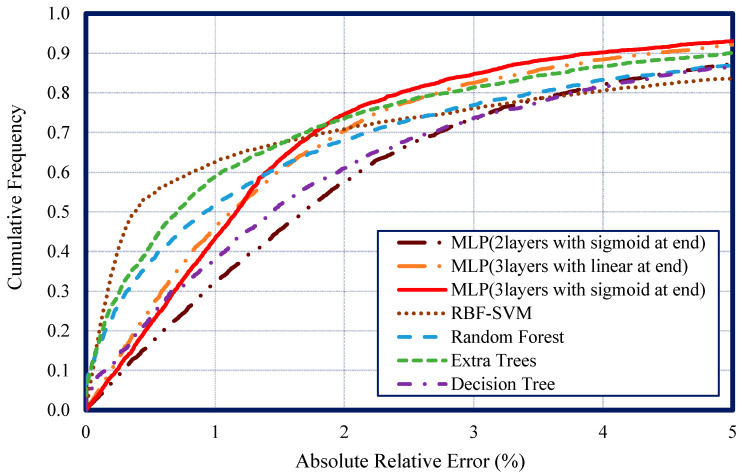
Cumulative frequency versus absolute relative error for all the mentioned models.

A better comparison of the presented models was done using the cumulative frequency as a function of absolute relative error (%) in Figure 5. As this figure shows, the MLP (with three layers and sigmoid at the end) could predict 85% of the data points with an absolute relative error of less than 3%, which outperformed all the models.

Moreover, from this figure, it can be seen that for the low absolute relative error (%), the RBF-SVM had the best performance where it could predict 63% of the data points with an absolute relative error less than 1%. The extra trees (ET) model showed consistent and acceptable performance in all absolute relative error ranges.

**Figure 6:**
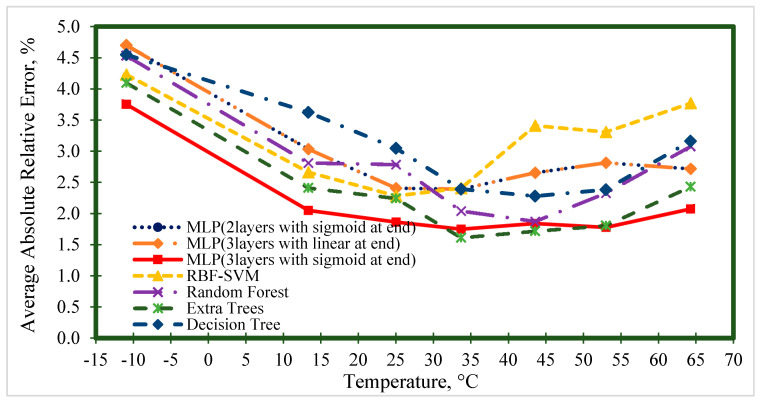
Average absolute relative error at different temperature ranges for prediction of the viscosity of nanofluids.

**Figure 7:**
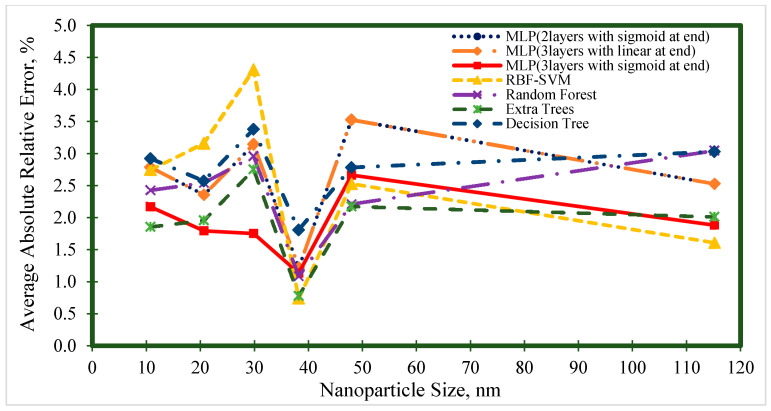
Average absolute relative error at different nanoparticle size ranges for prediction of the viscosity of nanofluids.

**Figure 8:**
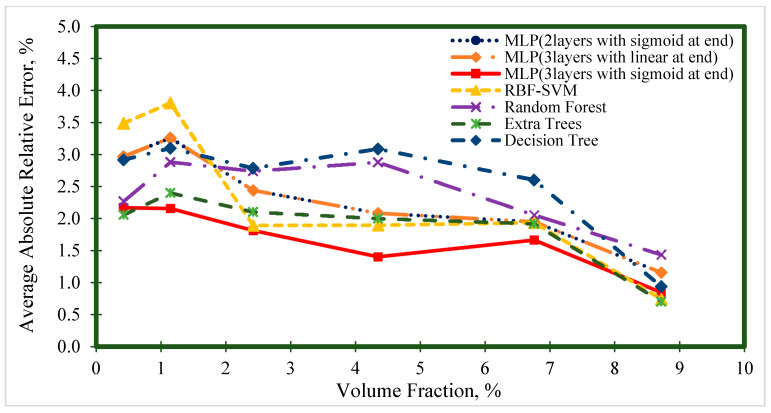
Average absolute relative error at different volume fraction ranges for prediction of the viscosity of nanofluids.

**Figure 9:**
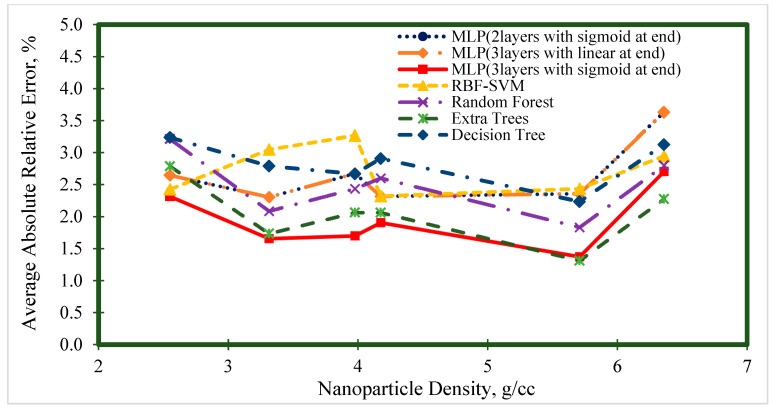
Average absolute relative error at different nanoparticle density ranges for prediction of the viscosity of nanofluids.

**Figure 10:**
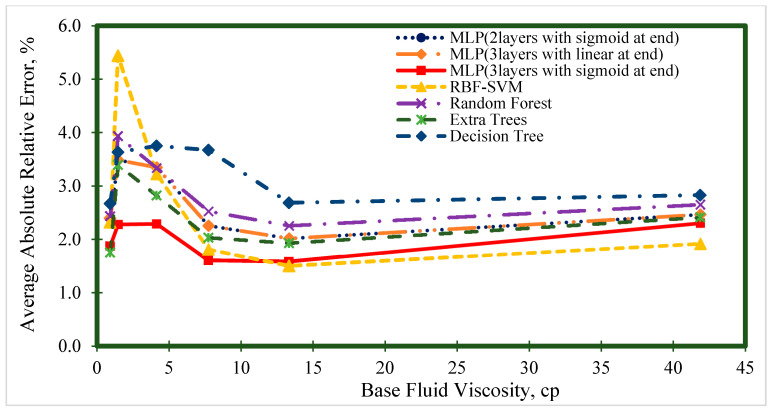
Average absolute relative error at different base fluid viscosity ranges for prediction of the viscosity of nanofluids.

Figure 6, Figure 7, Figure 8, Figure 9 and Figure 10 represent the trend of AARE (%) at different input ranges for all the presented models. In addition to determining the performance of each model by changing the input, these figures were used to specify which model was appropriate for a specific input range. For example, Figure 6 shows that the general trend of AARE (%) for all the models was decreasing by temperature until about 35 °C. It can be seen that for this temperature range (<35 °C), the MLP (with three layers and sigmoid at the end) had the best performance. For the other inputs, the MLP (with three layers and sigmoid at the end) showed almost the best performance.

**Figure 11:**
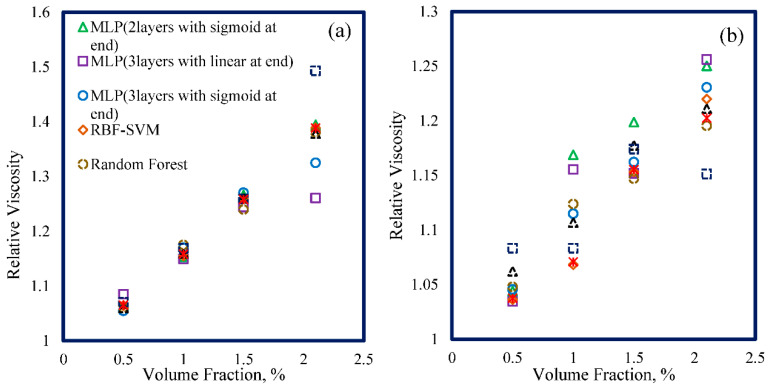
Variation of relative viscosity with a volume fraction of nanoparticles for two nanofluid samples, (**a**) ZnO (48 nm)—EG, (**b**) ZnO (4.6 nm)—EG [73].

To examine if the provided models can follow the physically expected trends of nanofluid viscosity by changing volume fraction, the predicted values by these models are represented in Figure 11. As can be seen in this figure, for two nanofluid samples, the experimental relative viscosity values increased with increasing the nanoparticles volume fraction. All the models could capture the expected trend with a variation of volume fraction, although some models had a slight deviation from the experimental data. This figure also proves that the proposed models were physically valid, with a variation of volume fraction as the most affecting parameter on the relative viscosity of nanofluids.

## 5. Conclusions

In this study, the viscosity of various nanofluids was modeled using advanced computational frameworks. To this end, eight machine learning models were proposed, including two multilayer perceptron (MLP), each with Nesterov accelerated adaptive moment (Nadam) optimizer; two MLP, each with three hidden layers and Adamax optimizer; a support vector regression (SVR) with radial basis function (RBF) kernel; a decision tree (DT); two tree-based ensemble models, including random forest (RF) and extra tree (ET). The data bank, which was used for modeling, includes 3144 data points of nanofluids at different volume fraction, size, and density of nanoparticles, temperature, and viscosity of base fluids. The performance of these models at different ranges of input variables was evaluated and compared with the literature models. Based on our result, all the eight suggested models outperformed the baselines used in the literature, and five of our presented models outperformed the CMIS model, where two of them returned an AARE less than 3% on the test data. In addition, the physical validity of models was confirmed by examining the physically expected trends of nanofluid viscosity due to changing volume fraction.

## Figures and Tables

**Table 1:** The data bank of nanofluids used in this study.

	Al_2_O_3_	CuO	SiO_2_	SiC	TiO_2_	Fe_3_O_4_	MgO	Mg(OH)_2_	Co_3_O_4_	Nanodiamond	ZnO
**References**	[36,37,38,39,40,41,42,43,44,45,46,47,48,49,50,51]	[39,49,52,53,54,55]	[41,56,57,58,59,60]	[61]	[36,44,46,47,62,63,64,65]	[66,67]	[24]	[68]	[69]	[70,71]	[72,73]
**Base fluid**	Water DI water Transformer oil R11 refigerant Polyalphaolefins EG EG/W 20:80 wt% EG/W 40:60 wt% EG/W 20:80 wt% W/EG 60:40 vol% W/EG 50:50 vol% W/EG 40:60 vol%	Water EG PG/W 30:70 vol% EG/W 60:40 wt%	Water Ethanol DI water Transformer oil EG EG/W 25:75% EG/W 50:50% BG/W 20:80 vol% BG/W 30:70 vol%	DI water	Water DI water EG EG/W 20:80 wt% BG/W 20:80 vol% BG/W 30:70 vol%	Water Toluene	EG	EG	EG	water EG/W 20:80 wt% EG/W 60:40 wt% EG/W 40:60 wt%	EG
**T (°C)**	0–72	−35–67	19–80	30	9.85–80	20–60	20–70	23–65	10–50	0–60	10–50
**φ (%)**	0.01–10	0–9	0–8.4	0–3	0.2–10	0.04–2	0.1–5	0.1–2	0.9–5.7	0.2–1	0.25–5
**d_p_ (nm)**	8–120	11–152	7–190	100	6–50	10–13	21–125	20	17	11.83–19.27	4.6–48
**ρ_P_ (gr/cm^3^)**	3.69–4	6.31	2.22–2.65	3.21	4.18–4.23	5.17–5.81	3.58	2.34	6.11	3.1	5.61–13.61
**μ_nf_ (cp)**	0.44–610.46	0.46–447.35	0.59–37.36	0.93–1.60	0.46–28.41	0.32–1.65	3.70–30.60	4.82–23.02	8.06–44.76	25.51	6.14–49.30
**μ_bf_ (cp)**	0.39–452.60	0.42–99.54	0.54–18.53	0.8	0.42–23.01	0.3–0.79	3.63–21.11	1.02–1.60	1.02–1.44	0.24–13.74	6.08–35.44
**No. of data points**	1197	500	278	5	308	121	198	35	25	357	122

W: Water, DI: Deionized, PG: Propylene Glycol, EG: Ethylene Glycol, BG: BioGlycol.

**Table 2:** Statistical error analysis for prediction of the relative viscosity of nanofluids.

Model		ARE (%)			AARE (%)			RMSE			SD	
	*Train*	*Test*	*Total*	*Train*	*Test*	*Total*	*Train*	*Test*	*Total*	*Train*	*Test*	*Total*
**CMIS [1]**	−0.382	−0.515	−0.409	3.933	4.036	3.954	0.094	0.088	0.093	0.062	0.061	0.062
**MLP [1]**: (5)(Tanh,12)(Sigmoid,8)(Linear,1)-BR	−0.440	−0.179	−0.387	4.557	4.931	4.632	0.100	0.113	0.103	0.069	0.074	0.070
**LSSVM [1]**: Optimized by CSA	−0.921	−1.029	−1.011	5.342	6.630	5.488	0.187	0.047	0.193	0.070	0.017	0.108
**MLP**: (5)(Tanh,32)(Sigmoid,64)(Linear,1)-Nadam	1.596	1.555	1.587	4.076	4.818	4.238	0.012	0.015	0.013	0.064	0.080	0.067
**MLP**: (5)(Tanh,32)(Sigmoid,64)(Sigmoid,1)-Nadam	−0.206	−0.457	−0.260	2.369	3.876	2.697	0.008	0.012	0.009	0.040	0.062	0.046
**RBF-SVM**: C = 1; gamma = 2.3	0.089	−0.131	0.041	2.120	4.740	2.690	0.010	0.023	0.014	0.051	0.096	0.064
**Decision Tree**: max depth = 14, max feature = 4, min samples split = 3, max leaf nodes = 450	−0.103	0.321	−0.011	2.043	4.579	2.595	0.005	0.022	0.011	0.032	0.087	0.050
**Random Forest**: max depth = 18, max feature = 4	−0.204	−0.499	−0.268	1.746	3.945	2.225	0.006	0.020	0.011	0.036	0.080	0.049
**Extra Trees**: max depth = 20, max feature = 5, min samples split = 4, max leaf nodes = 1000	−0.149	−0.335	−0.189	1.244	3.597	1.756	0.004	0.016	0.008	0.023	0.070	0.038
**MLP**: (5)(Tanh,64)(Sigmoid,128)(Sigmoid,16)(Linear,1)-AdaMax	1.063	1.181	1.088	1.632	2.914	1.911	0.005	0.010	0.006	0.030	0.051	0.036
**MLP**: (5)(Tanh,64)(Sigmoid,128)(Sigmoid,16)(Sigmoid,1)-AdaMax	−0.507	−0.635	−0.535	1.583	2.855	1.860	0.005	0.009	0.006	0.029	0.049	0.035
